# The role of TRPV4 in ischemic stroke

**DOI:** 10.1186/s13041-026-01274-6

**Published:** 2026-01-28

**Authors:** Juan Guo, Xiaotong Yu, Yuewen Ma

**Affiliations:** 1 Department of Rehabilitation Medicine, The First Affiliated Hospitalof China Medical University, Shenyang, 110001 China; 2 Institute of Meta-Synthesis Medicine, Beijing, 100097 China

**Keywords:** TRPV4, Ischemic stroke, Angiogenesis, Physical therapy

## Abstract

Recent advances in mechanotransduction research have highlighted the important role of mechanosensitive ion channels, particularly the transient receptor potential vanilloid 4 (TRPV4) channel. TRPV4, a non-selective cation channel predominantly located on the plasma membrane, is widely expressed in the mammalian and human brain and exhibits sensitivity to mechanical stimuli due to its unique structural features. Emerging evidence suggests that TRPV4 may function as a modulator in the pathophysiology of ischemic stroke. During the acute phase of stroke, TRPV4 activation has been linked to neuronal injury and cerebral edema. In contrast, during the recovery phase following ischemia-reperfusion, TRPV4 appears to contribute to neurovascular remodeling by facilitating intracranial arterial dilation and collateral vessel formation. These phase-dependent roles indicate that targeted modulation of TRPV4, particularly through physical therapies, could represent a potential therapeutic strategy to improve outcomes after ischemic stroke. This review summarizes current findings on TRPV4 in stroke pathobiology and discusses its potential as a mechanotherapeutic target.

## Introduction

Ischemic stroke is a major global health concern, primarily resulting from an interruption of cerebral blood flow that leads to acute and often irreversible neurological injury [1]. In 2016, an estimated 13.7 million new stroke cases were reported worldwide, with ischemic stroke accounting for approximately 87% of all cases [2]. In addition to its high incidence, ischemic stroke is associated with significant mortality, long-term disability, and a high risk of recurrence, placing considerable economic and psychological burdens on patients, families, and healthcare systems [1–4]. These challenges underscore the urgent need to better understand the underlying pathological mechanisms of ischemic stroke and to develop more effective therapeutic strategies.

In recent years, research has increasingly focused on the complex interplay among excitotoxicity, inflammation, angiogenesis, and neurogenesis during both the acute and chronic phases of ischemic brain injury [3, 5]. These processes are closely interrelated and can either amplify or counterbalance one another during disease progression [3]. Accordingly, identifying and characterizing molecular targets that mediate these interconnected pathways is essential for developing precise and mechanism-based interventions to improve clinical outcomes [5].

The transient receptor potential (TRP) channel family was first identified in 1969 in a Drosophila mutant exhibiting defective phototransduction [6]. Since then, TRP channels have been recognized as evolutionarily conserved polymodal sensors that detect a broad spectrum of physical and chemical stimuli and respond to tissue or cellular stress [7]. These multifunctional ion channels participate in a wide array of physiological and pathological processes and have been extensively investigated across multiple disciplines, including neurology, oncology, dermatology, pulmonology, cardiology, urology, pain research, and rare diseases [6, 7]. Recent studies have highlighted that several TRP channel subfamilies are implicated in the pathophysiology of ischemic stroke [7]. Although TRP channels share conserved structural motifs, they exhibit diverse gating mechanisms and biophysical properties. Their activation during cerebral ischemia can exert neuroprotective or neurotoxic effects in a context-dependent manner, influenced by the specific channel subtype, cellular localization, and phase of stroke progression [7].

The mammalian TRP channel superfamily comprises 28 members, which are classified into six subfamilies based on nucleotide sequence homology: TRPA (ankyrin), TRPC (canonical), TRPM (melastatin), TRPML (mucolipin), TRPP (polycystin), and TRPV (vanilloid) [8, 9]. Several TRP channels—including TRPC6, TRPM2, TRPV1, and TRPV4—have been implicated in the pathogenesis and progression of ischemic stroke, particularly through their roles in neurons and endothelial cells [10–19] (Table 1). Compared with other TRP subtypes, TRPV4 is uniquely characterized by its pronounced mechanosensitivity, which positions it as a promising candidate for studying the mechanistic basis and therapeutic potential of mechanical-based interventions in ischemic stroke [20]. Since its discovery in 2000, TRPV4 has been increasingly recognized for its involvement in key pathological processes such as neuronal injury, cerebral edema, and angiogenesis [17–19, 21–24]. Given its central role in mechanotransduction and neurovascular regulation, further elucidation of TRPV4-mediated molecular mechanisms and the development of physical therapies targeting its activity may contribute to more precise and effective strategies for ischemic stroke management.

## The structure and function of TRPV4

Structurally, TRPV4 consists of 871 amino acids and assembles as a homotetramer, with each subunit containing six transmembrane helices (S1–S6). Both the N- and C-terminal domains are located intracellularly, while the pore-forming loop lies between the S5 and S6 segments [25–27]. The channel exhibits relatively high calcium permeability, preferentially conducting Ca^2+^ ions over other cations such as Mg^2+^, K^+^, Cs^+^, Na^+^, and Li^+^ [28–30]. Two conserved aspartate residues (D672 and D682) within the pore region are important for determining ion selectivity and Ca^2+^ conductance [25–27]. In addition, the C-terminal domain of TRPV4 interacts with calmodulin (CaM) in a calcium-dependent manner, which influences conformational coupling between the N- and C-termini and contributes to the regulation of channel gating and downstream signaling pathways [29].

**Fig. 1:**
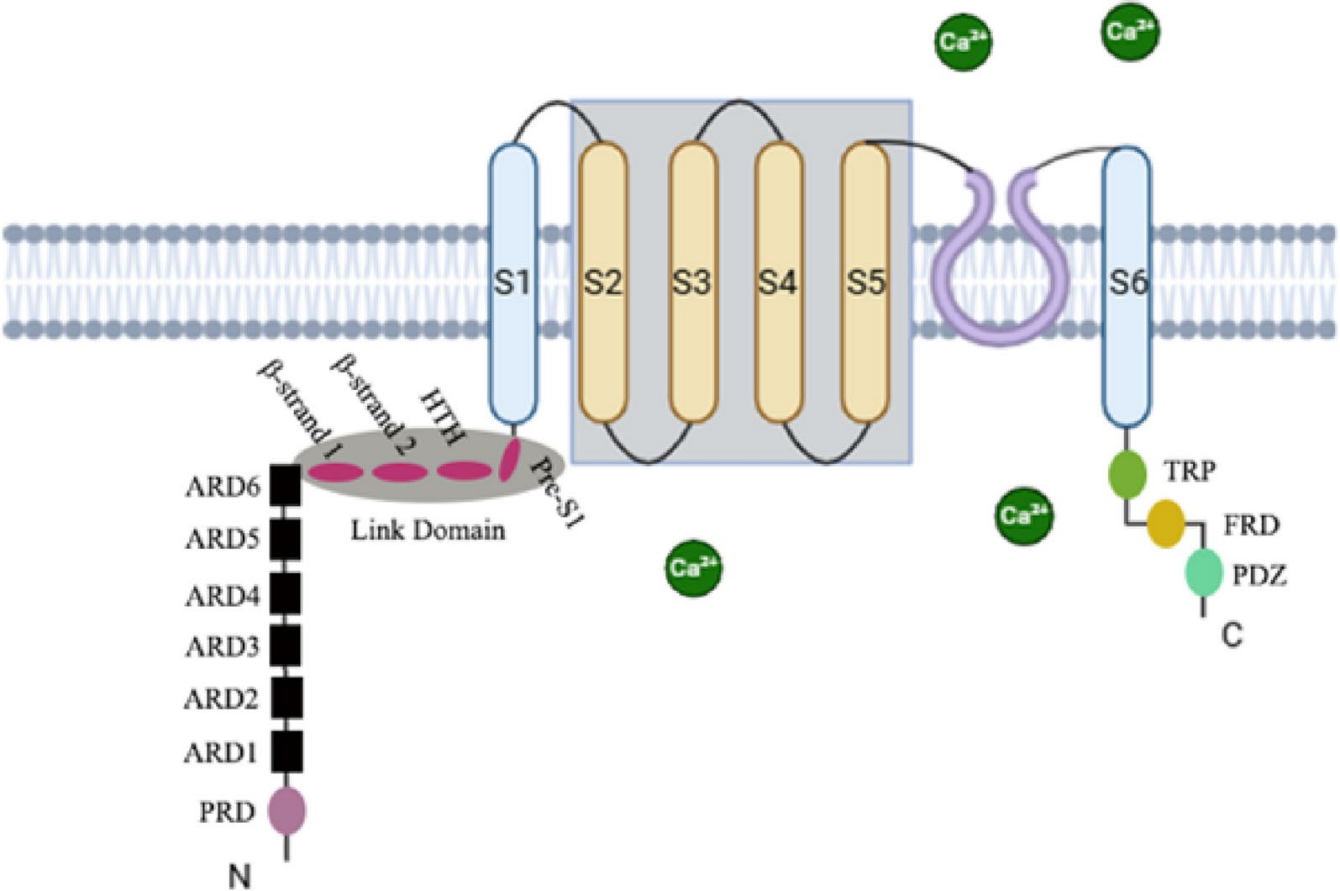
Main structural features of the TRPV4 protein: The TRPV4 channel is a tetrameric, non-selective cation channel composed of six transmembrane domains (S1–S6) and large cytoplasmic N- and C-terminal regions. The N-terminal region contains six ankyrin repeat domains (ARD1–ARD6), a PRD, and a linker domain connecting to the pre-S1 helix. The C-terminal region includes the TRP, the FRD, and the PDZ, which are involved in channel gating, membrane anchoring, and protein–protein interactions. The pore-forming loop between S5 and S6 allows Ca^2+^influx upon activation by mechanical, osmotic, or thermal stimuli

The TRPV4 channel can be activated by multiple physicochemical stimuli, including mechanical force, hypo-osmotic stress, temperature elevation, shear stress, and arachidonic acid metabolites [23, 31]. Its sensitivity to mechanical cues is associated with specific structural features that support its mechanogating mechanism [32]. The N-terminal region of TRPV4 contains several functional domains, including a proline-rich domain (PRD), six ankyrin repeat domains (ARDs), and a linker region composed of two β-strands, a helix-turn-helix (HTH) motif, and a pre-S1 helix. Following the N-terminus, an additional linker connects the transmembrane segments S3 and S4, while the C-terminus contains a conserved TRP domain (Fig. 1). These structural elements contribute to the regulation of channel gating in response to mechanical stimulation [33]. TRPV4 mechanosensitivity is further influenced by its interaction with the actin cytoskeleton. The ARDs within the N-terminal region can associate with actin filaments, allowing the channel to transmit mechanical inputs from the cellular environment and integrate them into ion channel activity and downstream signaling processes [34].

TRPV4 is expressed across a broad range of organ systems, including the brain, eyes, endocrine glands, respiratory and gastrointestinal tracts, liver, gallbladder, pancreas, kidneys, bladder, reproductive organs, muscle, connective tissue, skin, bone marrow, and lymphoid tissues [35]. Among these, relatively high expression levels have been reported in the brain, gastrointestinal tract, pancreas, and reproductive organs [35]. Within these tissues, TRPV4 is involved in various physiological processes, such as the modulation of neuronal excitability, detection of arterial shear stress and renal osmotic changes, and regulation of skeletal homeostasis [34, 36]. In the peripheral nervous system, TRPV4 is primarily localized in the dorsal root ganglia, where it has been shown to influence sensory neuron activity and contribute to nociceptive signaling [37, 38].

In the brain, TRPV4 is expressed in multiple cell types, including neurons, astrocytes, microglia, vascular endothelial cells, and smooth muscle cells (SMCs) of cerebral arteries [17]. By mediating calcium influx, TRPV4 participates in several intracellular signaling pathways, such as those involving nitric oxide (NO), phospholipase A2 (PLA2), extracellular signal-regulated kinase 1/2 (ERK1/2), calcium/calmodulin-dependent protein kinase II (CaMKII), and inositol 1,4,5-trisphosphate (IP3) [17, 31]. These signaling pathways are associated with a range of physiological and pathophysiological processes in the central nervous system. Accordingly, TRPV4 has been investigated in relation to several neurological disorders, including Alzheimer’s disease, ischemic stroke, traumatic brain injury, epilepsy, multiple sclerosis, and gliomas [39–42].

## The role of TRPV4 in ischemic stroke

### TRPV4 involvement in neuronal damage and cerebral edema formation

TRPV4 may play phase-dependent roles during ischemic stroke. In the acute ischemia-reperfusion phase, excessive TRPV4 activation has been associated with increased Ca^2+^ influx, which can disrupt cellular volume regulation and contribute to cerebral edema and neuronal injury [43]. Mechanistic studies indicate that TRPV4 knockout helps maintain blood-brain barrier (BBB) integrity, potentially via downregulation of matrix metalloproteinases (MMPs), suggesting a possible role for TRPV4 in early cerebral ischemia [44]. Pharmacological inhibition of TRPV4 with HC067047, administered intracerebroventricularly, has been reported to reduce neurological deficits in middle cerebral artery occlusion (MCAO) mice within 60 min post-infarction [45]. Further evidence indicates that TRPV4 activation during acute infarction contributes to neuronal injury through activation of GluN2 subunit-containing NMDA receptors and suppression of AKT signaling pathways [45]. Disruption of TRPV4-mediated Ca^2+^ homeostasis in neurons and astrocytes has been associated with increased cytotoxic swelling and neuroinflammation, which may exacerbate ischemic brain injury [46, 47]. For example, acute hypoxic stress has been shown to activate the PKA/arachidonic acid (AA)/TRPV4 signaling cascade in the prefrontal cortex, whereas selective TRPV4 blockade appears to reduce hypoxia-induced neuronal apoptosis [48]. Consistently, Öcal et al. reported that dual inhibition of TRPM2 and TRPV4 channels can mitigate mitochondrial oxidative stress, neuronal apoptosis, and neuroinflammatory responses under hypoxic conditions [49].

As ischemic injury resolves and cerebral edema subsides, potential neuroregenerative roles of TRPV4 have attracted attention. Moderate TRPV4 activation has been reported to increase the expression of vascular endothelial growth factor A (VEGFA) and endothelial nitric oxide synthase (eNOS), which may facilitate angiogenesis and neurorepair, potentially contributing to improved post-stroke functional outcomes [50]. In MCAO models, Reed et al. observed that administration of the TRPV4 agonist 4α-phorbol 12,13-didecanoate (4α-PDD) was associated with reduced infarct volume and enhanced motor function recovery [51].

Despite growing evidence for TRPV4’s involvement in fluid regulation and neurovascular remodeling, its precise roles in ischemic stroke remain incompletely understood [52, 53]. Conflicting results regarding whether TRPV4 activation or inhibition may be beneficial likely reflect its functional heterogeneity across brain cell types [46]. TRPV4 appears to have protective effects in endothelial cells, while it may contribute to injury in neurons and glial cells; however, this dichotomy is not absolute (Table 2). For example, Konno et al. reported that TRPV4 activation in microglia reduced lipopolysaccharide-induced TNF-α release, increased galectin-3 expression, and enhanced K^+^ current amplitude [54]. This finding suggests that TRPV4-induced depolarization could lower the extracellular Ca^2+^ driving force, potentially modulating microglial activation and neuroinflammatory responses. Similarly, in a collagenase-induced intracerebral hemorrhage model, the selective TRPV4 agonist GSK1016790A was associated with improved motor performance, particularly in the rotarod fatigue test, without evident changes in the number of Iba1-positive microglia/macrophages or in mRNA levels of pro-inflammatory cytokines such as IL-1β and IL-6. These observations underscore the context-dependent nature of TRPV4 signaling in the brain.

**Fig. 2:**
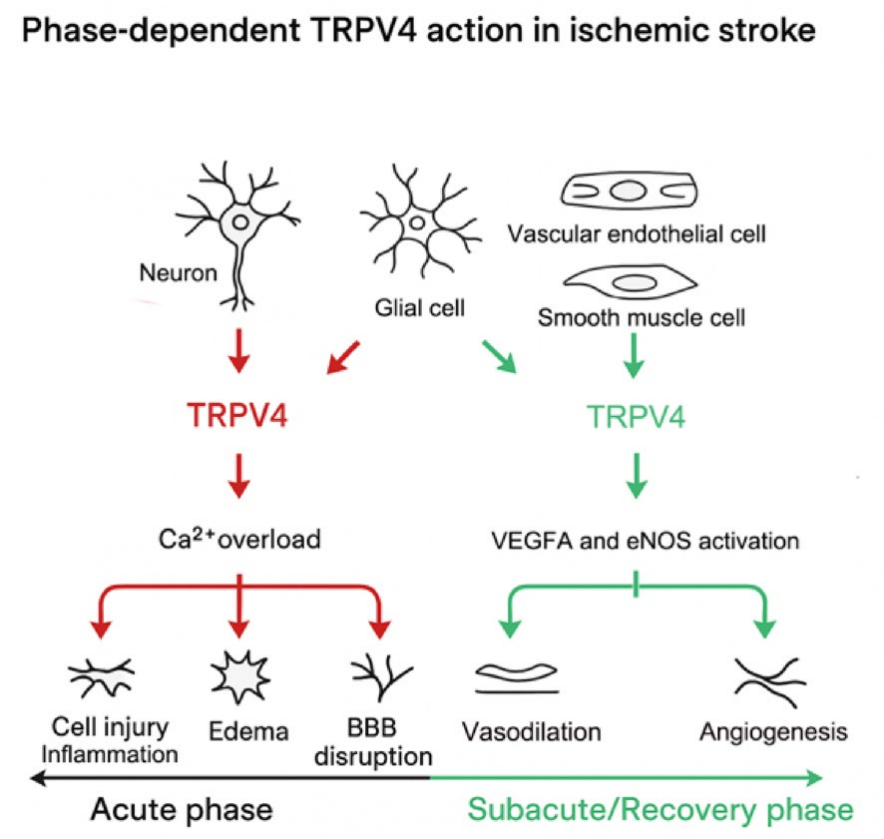
Phase dependent TRPV4 action in ischemic stroke: This schematic illustrates the dual roles of TRPV4 across different phases of ischemic stroke. During the acute phase, TRPV4 overactivation in neurons and glial cells leads to intracellular Ca^2+^ overload, contributing to cell injury, inflammation, edema, and BBB disruption. In contrast, during the subacute/recovery phase, TRPV4 activation in vascular endothelial and smooth muscle cells stimulates VEGFA and eNOS signaling, promoting vasodilation and angiogenesis, thereby enhancing neurovascular repair. These findings highlight the phase-dependent and cell-specific effects of TRPV4, emphasizing the need for temporally tailored therapeutic modulation in stroke management

Taken together, the dual—and sometimes opposing—roles of TRPV4 across different stroke phases and cell types highlight the complexity of its involvement in ischemic pathophysiology (Fig. 2). Further studies are needed to clarify its spatiotemporal functions and to inform the development of TRPV4-targeted interventions. In the following sections, we focus on the mechanistic roles of TRPV4 in cerebral blood flow regulation and examine its potential therapeutic relevance.

### The role of TRPV4 in improving cerebral blood flow

The potential beneficial effects of TRPV4 activation under ischemic conditions are largely attributed to its involvement in vascular regulation [55–59]. Mechanical stimuli, such as shear stress, serve as key activators of TRPV4, which in turn modulates vascular function through multiple mechanisms [60]. First, TRPV4 interacts with β1-integrin, acting as a scaffold to regulate the localization of β-catenin at adherens junctions. Second, TRPV4 activation can influence RhoA activity, which regulates cytoskeletal dynamics and endothelial cell migration. Sustained RhoA activation may facilitate cytoskeletal reorganization and alter endothelial membrane permeability, potentially contributing to improved local blood perfusion in ischemic tissues [60].

In addition to its vascular roles, TRPV4 is involved in neuronal and glial signaling and may affect cerebral blood flow by modulating vascular tone in smooth muscle cells under mechanical stress [61]. In cerebrovascular endothelial cells, TRPV4 appears to be important for cell migration and arterial vasodilation [27, 61]. In astrocytes, TRPV4 channels can be activated by mechanical stimuli and may participate in microvascular blood flow regulation via the TRPV4–cyclooxygenase-1 (COX-1) signaling pathway [62].

Beyond the brain, TRPV4 activation has been associated with improved perfusion in other ischemic models, including hindlimb ischemia, myocardial ischemia, and tissue flaps in reconstructive surgery [55–57]. In the cerebral circulation, magnetic resonance imaging (MRI) studies indicate that TRPV4 inhibition is associated with reduced cerebral blood perfusion in rats, highlighting its role in maintaining cerebral hemodynamics [58]. Mechanistically, TRPV4 may promote SMCs remodeling in response to mechanical stress, thereby facilitating vasodilation, and it may also stimulate cerebrovascular endothelial cell proliferation through a Ca^2+^/calmodulin-dependent protein kinase (CaMK)-mediated pathway, supporting angiogenesis and potentially enhancing tissue perfusion following ischemic injury [63].

#### Vasodilation

Activation of TRPV4 channels has been reported to induce cerebral vasodilation, primarily in the arterial system [64, 65]. Several studies suggest that TRPV4 contributes to acetylcholine-induced vasodilation, with nitric oxide (NO) acting as a key regulatory mediator [66, 67]. For instance, Köhler et al. demonstrated that shear stress stimulates endothelium-dependent vasodilation in rat carotid arteries via the TRPV4/eNOS/NO signaling axis, an effect that was abolished by TRPV4 antagonists [68]. Interestingly, the interaction between TRPV4 and NO appears to be bidirectional: while TRPV4 activation can promote NO production and subsequent vasodilation [69], NO generated through the TRPV4/eNOS pathway may activate a guanylate cyclase (GC)/protein kinase G (PKG)-dependent negative feedback loop, potentially reducing TRPV4 channel activity in endothelial cells [64].

Beyond larger arteries, TRPV4 may also influence NO production in capillaries, where it can regulate local blood flow through Ca^2+^-dependent interactions with pericytes [70]. Cyclooxygenase (COX) pathways may further contribute to TRPV4-mediated vasodilation; notably, even when both COX activity and NO synthesis are inhibited (via L-NAME), TRPV4 activation can still elicit endothelium-dependent vasodilation, suggesting involvement of additional mediators [71].

Mechanistically, TRPV4-induced Ca^2+^ influx initiates a cascade of intracellular signaling events that may culminate in vasodilation [72]. In endothelial cells, TRPV4 activation is associated with a sustained increase in cytosolic Ca^2+^^2+^, enhancing PLC activity, leading to phosphatidylinositol 4, 5-bisphosphate (PIP2) depletion and IP3 production [72]. IP₃ subsequently stimulates Ca^2+^ release from intracellular stores, amplifying the vasodilatory response. Marrelli et al. reported that PLA2 inhibition attenuates both the TRPV4-induced Ca^2+^ rise and uridine triphosphate (UTP)-induced vasodilation in rat middle cerebral artery endothelial cells [73].

In vascular tissues, TRPV4 can also be activated by mechanical forces, contributing to carotid artery dilation via combined Ca^2+^ signaling and endothelium-derived hyperpolarizing factor (EDHF) [74]. Importantly, TRPV4-mediated vasodilation is not restricted to endothelial cells. In vascular smooth muscle cells, TRPV4 activation can facilitate Ca^2+^ influx, which may trigger Ca^2+^ release from the sarcoplasmic reticulum [75]. This signaling cascade can activate large-conductance calcium-activated potassium (KCa1.1) channels, resulting in membrane hyperpolarization and smooth muscle relaxation [75].

**Fig. 3:**
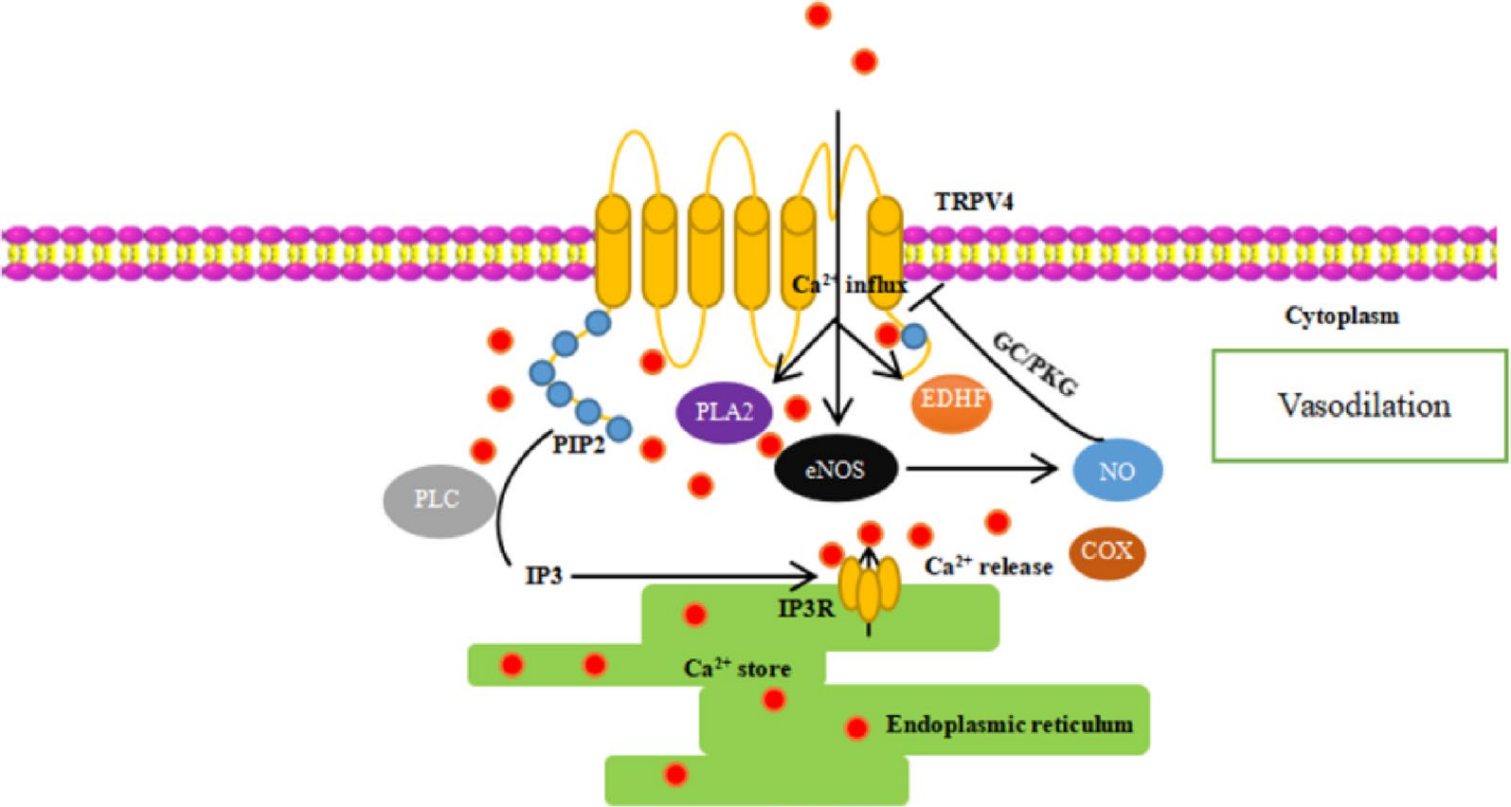
Schematic illustration of the TRPV4-mediated endothelial-dependent vasodilation pathway.: Activation of TRPV4 channels induces Ca^2+^ influx, which stimulates eNOS-derived NO production and subsequent vasodilation via the cGMP–PKG pathway. Concurrently, TRPV4-mediated Ca^2+^ signaling activates PLA2 and PLC–IP3 pathways, generating EDHF and prostanoids that further enhance vascular relaxation. Together, these Ca^2+^-dependent mechanisms maintain endothelial function and vascular homeostasis

In summary, TRPV4 activation engages a complex network of Ca^2+^-dependent signaling pathways involving PLA2, PLC, IP3, NO, COX, and EDHF. These pathways collectively contribute to cerebral vasodilation through coordinated actions in both endothelial and smooth muscle cells [59, 60, 68, 76] (Fig. 3).

#### Angiogenesis

Mechanical stimulation–induced activation of TRPV4 has been reported to play a role in angiogenesis [77]. Accumulating evidence suggests that TRPV4 may facilitate endothelial cell proliferation and migration in response to biomechanical cues, such as shear stress [63]. For example, Troidl et al. demonstrated that TRPV4 expression in endothelial cells is upregulated in a flow-dependent manner; exposure to laminar shear stress (8 dyne/cm²) increased TRPV4 mRNA and protein levels, which was associated with enhanced endothelial proliferation and the initiation of collateral vessel formation [63].

Mechanistically, TRPV4 may contribute to cytoskeletal reorganization and modulate cell adhesion dynamics, supporting endothelial cell division and directional migration during angiogenesis [78]. Some studies indicate that these effects involve the Rho/Rho kinase pathway, which regulates actin cytoskeleton remodeling and cell motility [79]. In addition to structural roles, TRPV4-mediated Ca^2+^ influx has been reported to stimulate the production and secretion of pro-angiogenic molecules such as vascular endothelial growth factor (VEGF) and platelet-derived growth factor (PDGF), potentially enhancing neovascularization [80, 81].

Moreover, pro-angiogenic factors including VEGF and basic fibroblast growth factor (bFGF) may further activate TRPV4, establishing a positive feedback loop that reinforces calcium-dependent signaling and supports vascular sprouting [82]. Conversely, pharmacological blockade of Ca^2+^ influx has been shown to inhibit VEGF-induced tube formation, highlighting the potential importance of TRPV4-mediated Ca^2+^ signaling in endothelial angiogenic responses [82]. TRPV4 activation has also been associated with upregulation of eNOS, which may promote angiogenesis and arteriogenesis, i.e., remodeling of pre-existing vessels into functional arteries [83].

Supporting these observations, activation of TRPV4 with the selective agonist 4α-PDD induces Ca^2+^ influx in human brain capillary endothelial cells and is associated with increased proliferation. This effect is attenuated by TRPV4-specific siRNA, consistent with a functional role for TRPV4 in cerebral angiogenesis [76].

## The impact of physical therapy on ischemic stroke through TRPV4

### Improvement of inflammation and cerebral edema

In the early stages of ischemic stroke, TRPV4 has been implicated as an ion channel involved in inflammatory responses and the development of cerebral edema. Upon activation, TRPV4 may contribute to neuroinflammation and fluid imbalance, potentially exacerbating ischemic brain injury. Several studies suggest that pharmacological or physical modulation of TRPV4 can attenuate ischemia-induced inflammation and reduce cerebral edema [84]. For example, electroacupuncture at the Shuigou (GV26) and Baihui (GV20) acupoints in MCAO rats has been reported to influence TRPV4 activity in microglia/macrophages, suppress M1 polarization, and reduce neuroinflammatory responses, thereby conferring neuroprotective effects [84].

Early treadmill exercise has also been associated with alleviation of cerebral edema through modulation of the caveolin-1/TRPV4/aquaporin-4 (AQP4) signaling axis, suggesting a mechanistic link between TRPV4 regulation and astrocytic water transport [85]. More recently, low-intensity pulsed ultrasound (LIPUS) has been reported to enhance glymphatic influx via TRPV4/AQP4-dependent mechanisms in astrocytes, providing a non-invasive approach to promote interstitial fluid clearance and reduce edema [86].

Collectively, these findings indicate that TRPV4 may serve as a potential target for modulating early neuroinflammatory and edema-related responses in ischemic stroke. Further studies are needed to clarify the underlying pathways and to optimize physical or pharmacological strategies for targeted intervention in post-stroke brain injury.

### Improvement of blood circulation

Optimizing cerebral perfusion is critical for salvaging viable brain tissue following ischemic stroke and supporting neurological recovery [87]. Various physical therapies have been reported to enhance cerebral blood flow post-stroke, which may contribute to functional improvement [88, 89]. These effects are thought to involve modulation of angiogenesis-related signaling pathways [90]. Key mediators in cerebral circulatory enhancement include hypoxia-inducible factor 1-alpha (HIF-1α), VEGF, BDNF, stromal cell-derived factor 1 (SDF-1), eNOS, and angiopoietin-1 (Ang-1) [91]. Notably, both HIF-1α and eNOS have been identified as downstream targets of the mechanosensitive ion channel TRPV4, suggesting a potential role for TRPV4 in mechanotransduction-mediated vascular remodeling [92, 93].

As a membrane-bound mechanosensor, TRPV4 may contribute to the effects of various physical modalities aimed at improving perfusion in ischemic brain regions. For instance, massage therapy—commonly used for musculoskeletal disorders—has been reported to enhance microcirculation, although the precise mechanisms remain unclear [94]. One proposed mechanism is that mechanical shear forces during massage stimulate mast cell degranulation and histamine release, which may enhance local blood flow [94]. Supporting this, Yang et al. showed that TRPV channel inhibitors can block shear stress–induced histamine release, implicating TRPV4 as a potential mediator of massage-induced circulatory effects [94]. Exercise training also appears to influence vascular function through TRPV4. By facilitating endothelial sensing of shear stress, TRPV4 channels may mediate the transmission of mechanical cues across the cardiovascular system, contributing to the regulation of vascular tone and hemodynamics [95]. Emerging evidence suggests that exercise can restore TRPV4-KCa2.3 signaling in aged arteries, potentially reversing age-related impairment of EDHF-mediated vasodilation [96].

Furthermore, LIPUS and extracorporeal shock wave therapy (ESWT)—two emerging mechanical rehabilitation modalities—may exert part of their biological effects via TRPV4 activation. Given TRPV4’s role in vascular tone regulation, endothelial function, and angiogenesis, it may serve as a key molecular mediator underlying the pro-circulatory and neuroprotective effects of these interventions.

#### LIPUS

LIPUS is a neuromodulation approach characterized by its safety, non-invasiveness, deep tissue penetration, and high spatial precision, enabling millimeter-scale targeting [97]. LIPUS has been investigated for its potential therapeutic effects in various cranial disorders, including ischemic stroke [97–99]. Mechanistically, LIPUS is proposed to act through activation of mechanosensitive ion channels, such as Piezo and TRPV4, thereby modulating neuronal and glial cell function [100]. In addition to its neuroregulatory effects, LIPUS has been reported to promote angiogenesis in brain-derived endothelial cells [101, 102]. These effects are thought to result from mechanical and cavitational stimulation, which may enhance the expression and release of neurotrophic and angiogenic factors, including BDNF and VEGF, contributing to neuroprotection and vascular remodeling [103].

Most studies examining the relationship between LIPUS and TRPV4 have focused on chondrogenesis in osteoarthritis models or signal modulation in retinal ganglion cells [104, 105]. Recent evidence suggests that LIPUS can increase cerebral blood flow in the motor cortex, potentially through preservation of endothelial integrity and enhancement of microvascular density, which may involve TRPV4-dependent signaling [106]. In experimental stroke models, Ichijo et al. reported that LIPUS stimulation (32 cycles, energy density of 193 mW/cm²) applied to the ischemic hemisphere in MCAO mice increased the number of CD31-positive vessels around the infarct, suggesting a pro-angiogenic effect [107]. Similarly, Nishida et al. observed that LIPUS-induced TRPV4 activation led to phosphorylation of p38 MAPK and ERK1/2, which could regulate VEGFR2 signaling, a pathway involved in angiogenesis [108, 109]. These findings indicate that LIPUS may influence neurovascular processes after ischemic injury, highlighting its potential utility in strategies aimed at supporting vascular repair and functional recovery.

#### ESWT

ESWT is a non-invasive modality that exerts biological effects primarily through mechanotransduction. It has been investigated for its potential to promote tissue repair and vascular regeneration in both clinical and experimental settings [110–113]. Owing to its non-invasive nature, ESWT has been applied in conditions affecting blood circulation, including ischemic stroke, spinal cord injury, ischemic heart disease, avascular necrosis of the femoral head, erectile dysfunction, and chronic wounds [114–119]. In central nervous system disorders, preclinical studies have shown that ESWT can support functional recovery in spinal cord injury models, enhancing both motor and sensory outcomes [119, 120].

Mechanistically, ESWT is proposed to facilitate angiogenesis and upregulate VEGF expression, contributing to vascular remodeling and potentially supporting neuronal survival [119, 120]. In ischemic stroke models, ESWT has been associated with activation of neurovascular repair processes, including stimulation of vascular and neural stem cell regeneration [121–123]. Some studies have indicated that ESWT promotes cerebrovascular angiogenesis in MCAO rats via activation of the Wnt/β-catenin signaling pathway [124]. Emerging evidence also suggests that ESWT can activate TRPV4 channels in the brain, potentially linking mechanical stimulation to calcium-dependent signaling pathways involved in cerebral perfusion and neuroprotection [125]. These findings suggest that ESWT may influence neurovascular function after ischemic injury; however, further studies are required to clarify the underlying mechanisms and to optimize parameters for TRPV4-targeted interventions.

## Limitation

Most evidence regarding TRPV4 in ischemic stroke comes from animal or in vitro studies, and human data remain very limited. While TRPV4 expression has been observed in experimental ischemic tissue, there are few studies examining its levels or functional correlates in stroke patients. Similarly, clinical trials targeting TRPV4 or other TRP channels are scarce, and none have yet directly investigated stroke. These gaps highlight the need for future research to quantify TRPV4 in human post-stroke brain, CSF, or peripheral biomarkers, correlate expression with imaging markers of perfusion and edema, and evaluate the safety and efficacy of TRPV4-modulating interventions in early-phase clinical studies. Consequently, the limited clinical evidence represents a key limitation of current understanding and underscores the need for translational studies to confirm preclinical findings.

## Prospect

TRPV4 plays multifaceted roles in ischemic stroke, including the regulation of neuroinflammation, maintenance of blood–brain barrier integrity, and promotion of angiogenesis and microcirculatory perfusion. Physical therapies that modulate TRPV4—such as LIPUS, ESWT, and electroacupuncture—hold considerable therapeutic potential. Future studies should clarify the interplay between TRPV4 and other signaling pathways to enable precise, targeted interventions. Optimizing therapy protocols—including timing, dosage, frequency, and potential combination strategies—will be essential to maximize efficacy while ensuring safety. For instance, Positive feedback loops between TRPV4 and pro-angiogenic factors such as VEGF present a therapeutic opportunity in ischemic stroke. Timed, moderate activation of TRPV4 during the subacute/recovery phase, using pharmacological agonists (e.g., 4α-PDD) or controlled physical therapies, could enhance VEGF-mediated angiogenesis and neurovascular repair. To achieve both efficacy and safety, such interventions should be guided by biomarkers of perfusion, BBB integrity, and cerebral edema (e.g., MRI perfusion/ADC, serum MMP-9, or VEGF levels), with careful titration of dosage, frequency, and duration.

In summary, TRPV4 represents a critical regulatory hub in ischemic stroke pathogenesis and recovery. Continued exploration of its mechanistic roles and translational potential may open new avenues for personalized and non-invasive stroke treatment and rehabilitation.

**Table 1:** Roles of different TRP channels in ischemic stroke and their functions in neurons and endothelial cells

Channel type	Main cell types	Role in ischemic stroke	Functions in neurons	Functions in endothelial cells	Representative references
TRPC6	Neurons and neurovascular cells	Bidirectional, mainly neuroprotective	Inhibits NMDA receptor–mediated excitotoxicity; overexpression might aggravate neuronal death	Mediates Ca^2+^ influx affecting endothelial hyperpolarization and cerebral blood flow regulation	[10, 11]
TRPM2	Neurons, glia, and endothelial cells	Mainly detrimental	Activated by oxidative stress, leading to neuronal death	Promotes ischemia-induced endothelial injury	[12–14]
TRPV1	Neurons and non-neuronal cells	Bidirectional, mainly protective	Mediates autophagy to reduce inflammation; overactivation might worsen neuron injury	Induces endothelium-dependent vasodilation via NO, prostacyclin, and K^+^ channels	[15, 16]
TRPV4	Neurons, glia, endothelial and smooth muscle cells	Bidirectional	Enhances glutamate release and excitotoxicity; might elevate neuronal activity to enhance brain injury recovery	Promotes Ca^2+^-dependent eNOS activation, mitochondrial activity, and angiogenesis	[17–19]

**Table 2:** Cell- and Phase-Specific roles of TRPV4 activation in ischemic stroke

Cell type	Effect of TRPV4 activation	Key signaling pathway / mechanism	Stroke phase	Representative references
Neuron	Generally harmful: excessive activation causes pathological Ca^2+^ influx, mitochondrial oxidative stress, and apoptosis	Ca^2+^ overload → GluN2-NMDA receptor activation → AKT inhibition; PKA/AA/TRPV4 cascade under hypoxia	Acute phase	[44, 46, 49, 50]
Astrocytes	Contributes to cytotoxic edema and neuroinflammation through Ca^2+^-dependent swelling	TRPV4-mediated Ca^2+^ influx → AQP4 and inflammatory signaling (e.g., STAT3)	Acute phase	[47, 48]
Microglia	Dual effects: generally pro-inflammatory but may suppress TNF-α release under certain conditions	TRPV4-induced depolarization reduces extracellular Ca^2+^ driving force → dampens overactivation	Acute–subacute phases	[55]
Endothelial cells	Protective: moderate activation promotes angiogenesis and neurorepair; knockout preserves BBB integrity	Ca^2+^ → eNOS / VEGFA / MMP regulation	Recovery phase (angiogenesis and remodeling)	[45, 51, 52]
Smooth muscle cells	Regulates vascular tone and contributes to perfusion recovery; excessive activation may cause edema	Ca^2+^-dependent myogenic and SK/IK channel signaling	Acute and recovery phases	[53, 54]

## Data Availability

No datasets were generated or analysed during the current study.
